# Supplementary figures and images for: Effects of poly (ADP-ribose) polymerase-1 (PARP-1) inhibition on sulfur mustard-induced cutaneous injuries in vitro and in vivo
Source: PeerJ. 2016 Apr 4;4:e1890. doi: 10.7717/peerj.1890 (PMC4830333; doi:10.7717/peerj.1890)

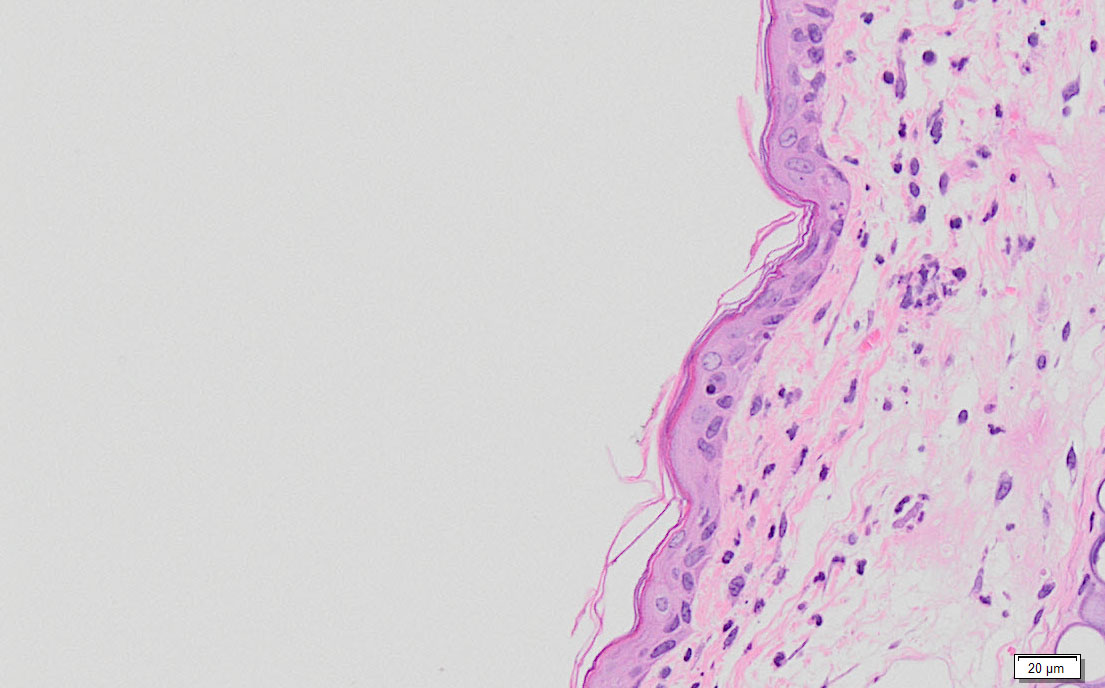

Supplement: Figure S1 [file peerj-04-1890-s003.zip › Raw data for Fig 3 A/0.16mg SM+ABT888-1.jpg]

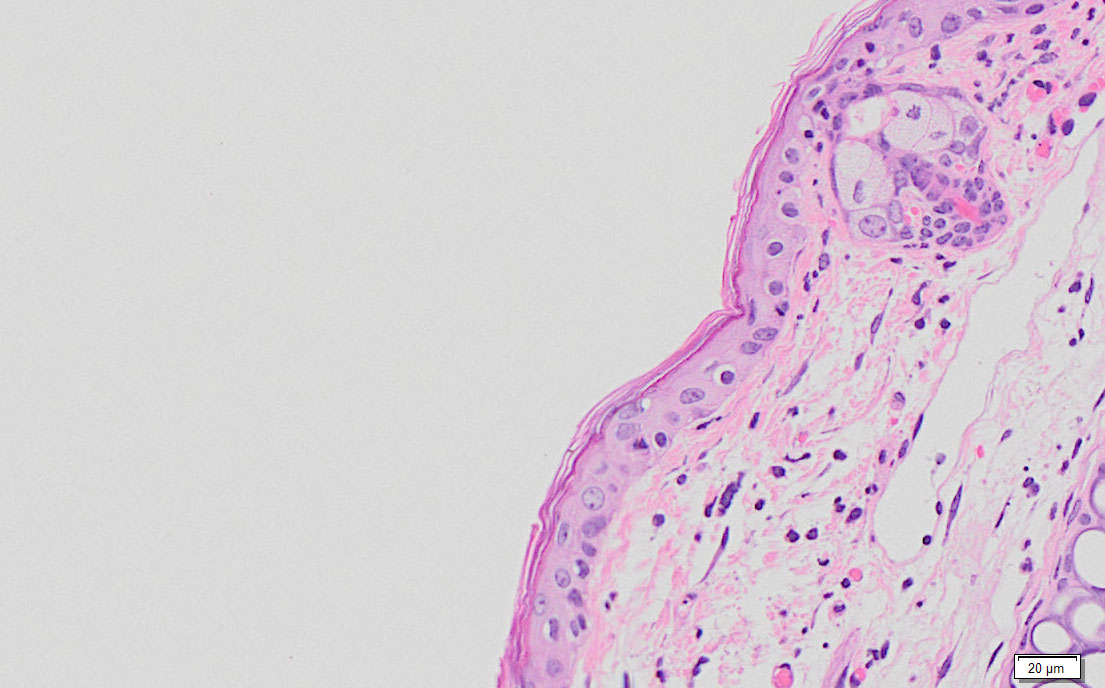

Supplement: Figure S1 [file peerj-04-1890-s003.zip › Raw data for Fig 3 A/0.16mg SM+ABT888-2.jpg]

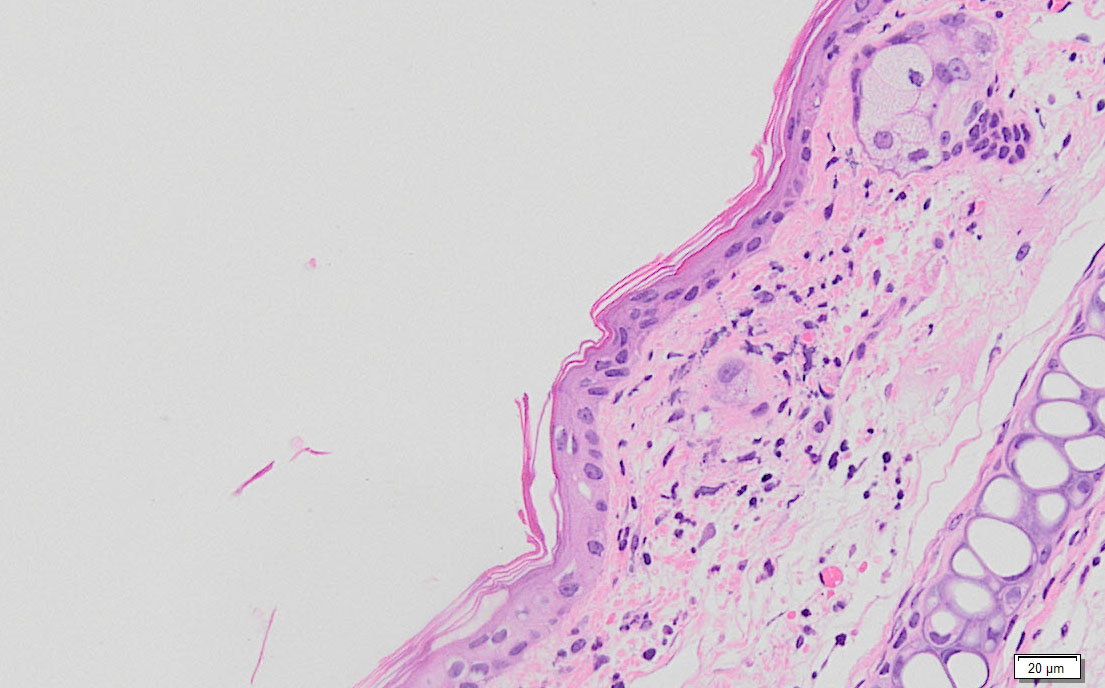

Supplement: Figure S1 [file peerj-04-1890-s003.zip › Raw data for Fig 3 A/0.16mg SM+ABT888-3.jpg]

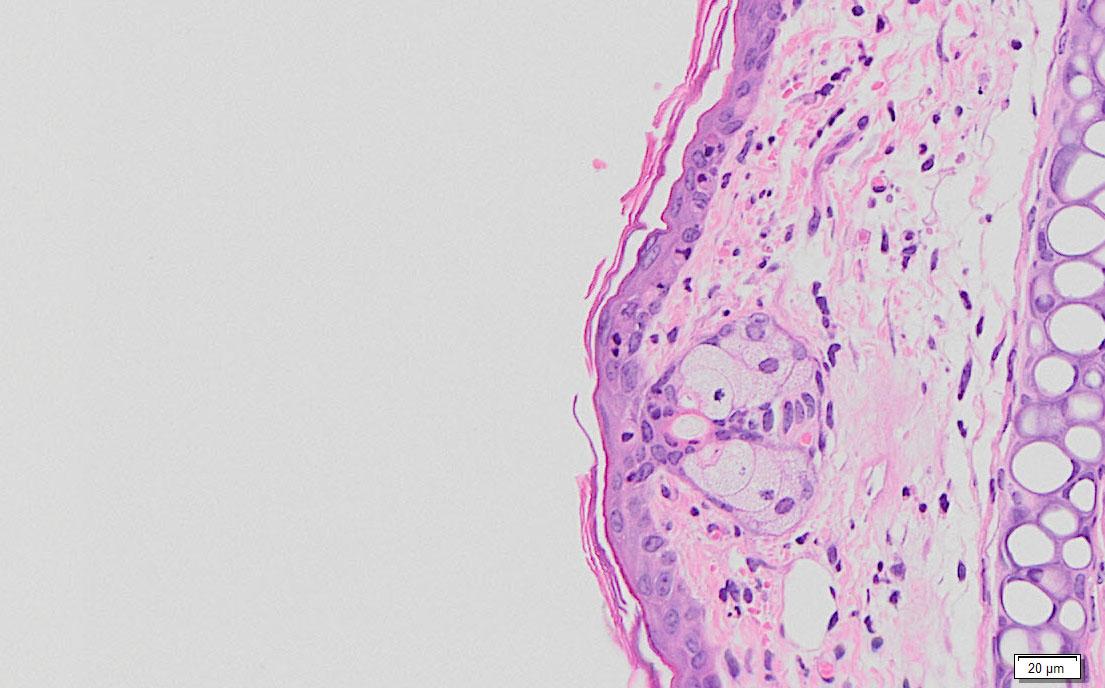

Supplement: Figure S1 [file peerj-04-1890-s003.zip › Raw data for Fig 3 A/0.16mg SM+ABT888-4.jpg]

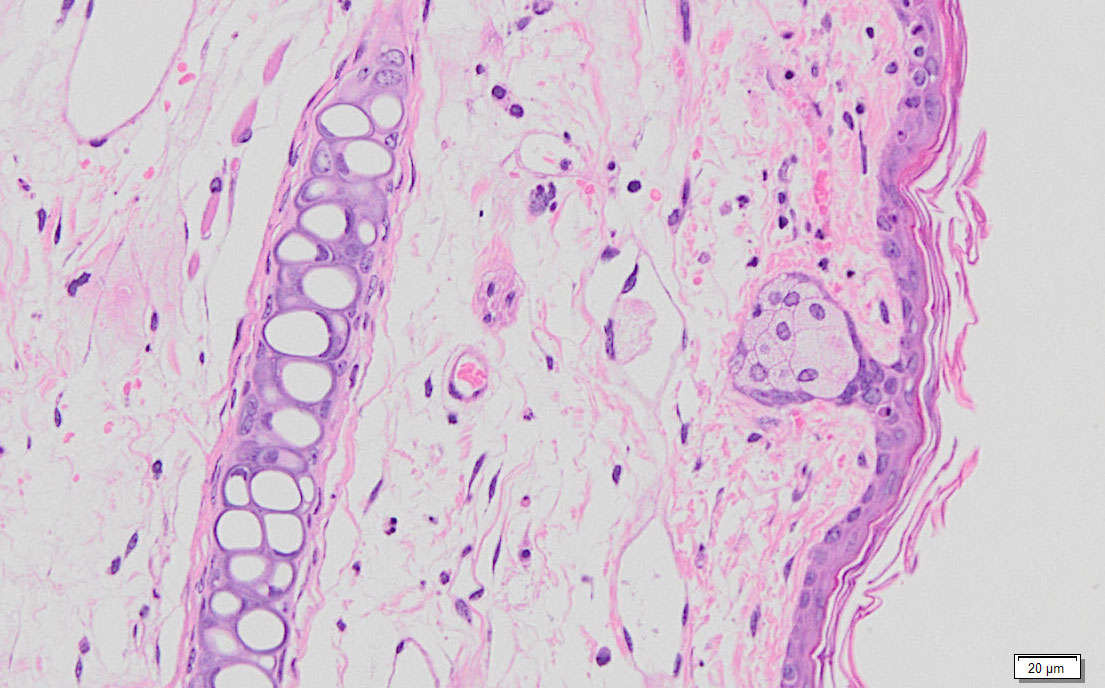

Supplement: Figure S1 [file peerj-04-1890-s003.zip › Raw data for Fig 3 A/0.16mg SM+ABT888-5.jpg]

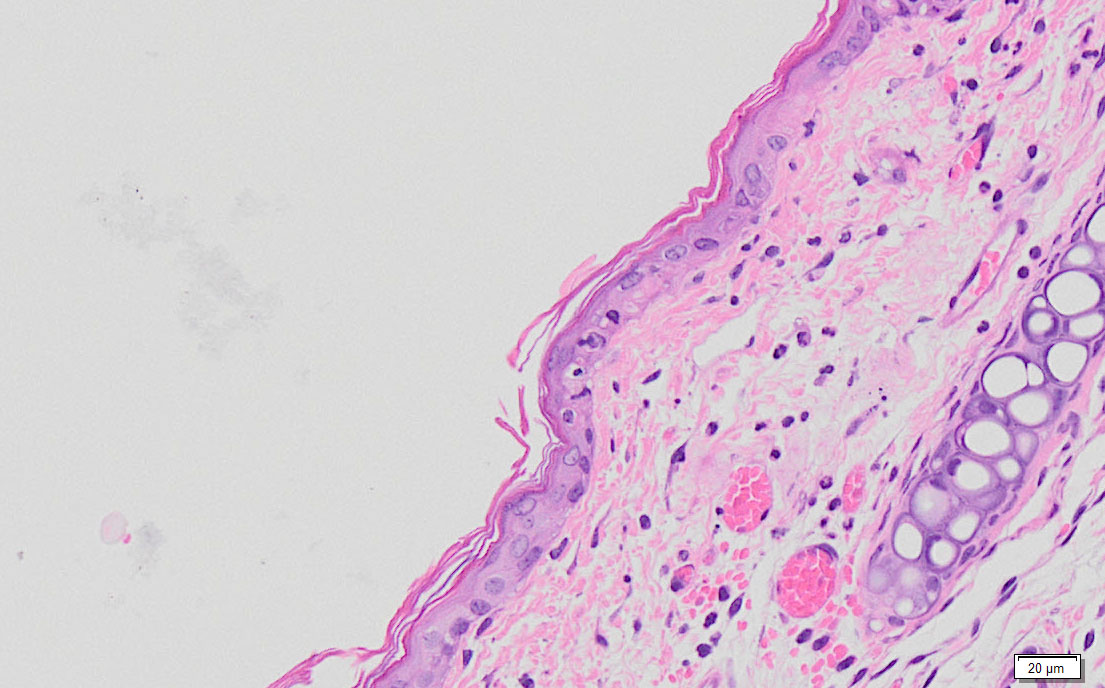

Supplement: Figure S1 [file peerj-04-1890-s003.zip › Raw data for Fig 3 A/0.16mg SM-1.jpg]

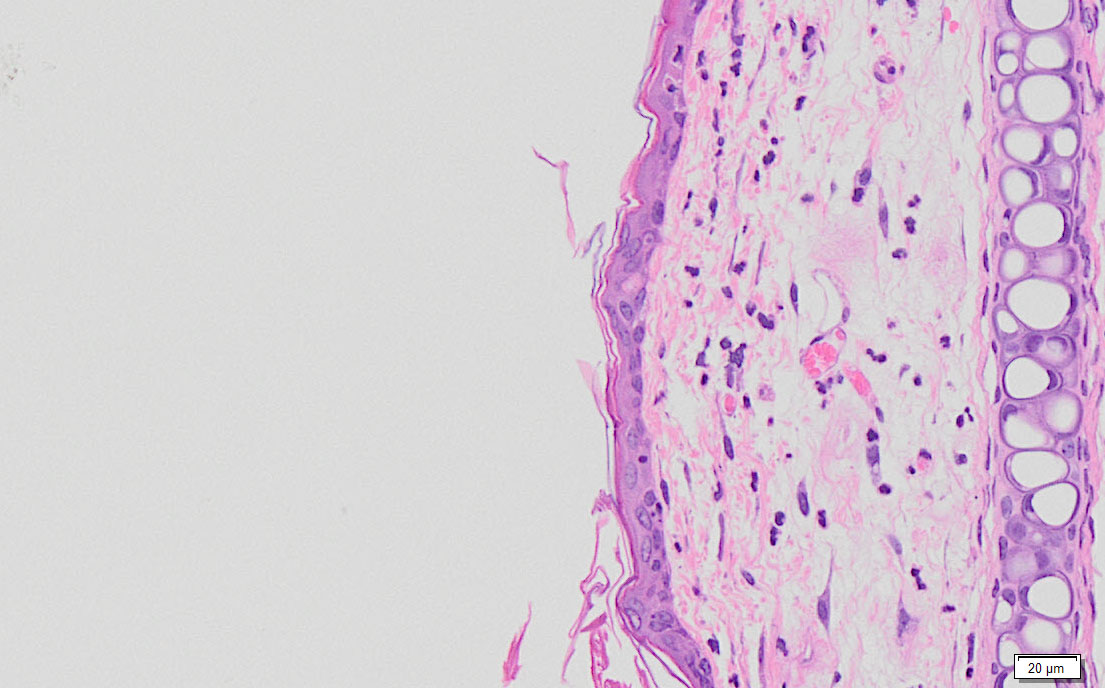

Supplement: Figure S1 [file peerj-04-1890-s003.zip › Raw data for Fig 3 A/0.16mg SM-2.jpg]

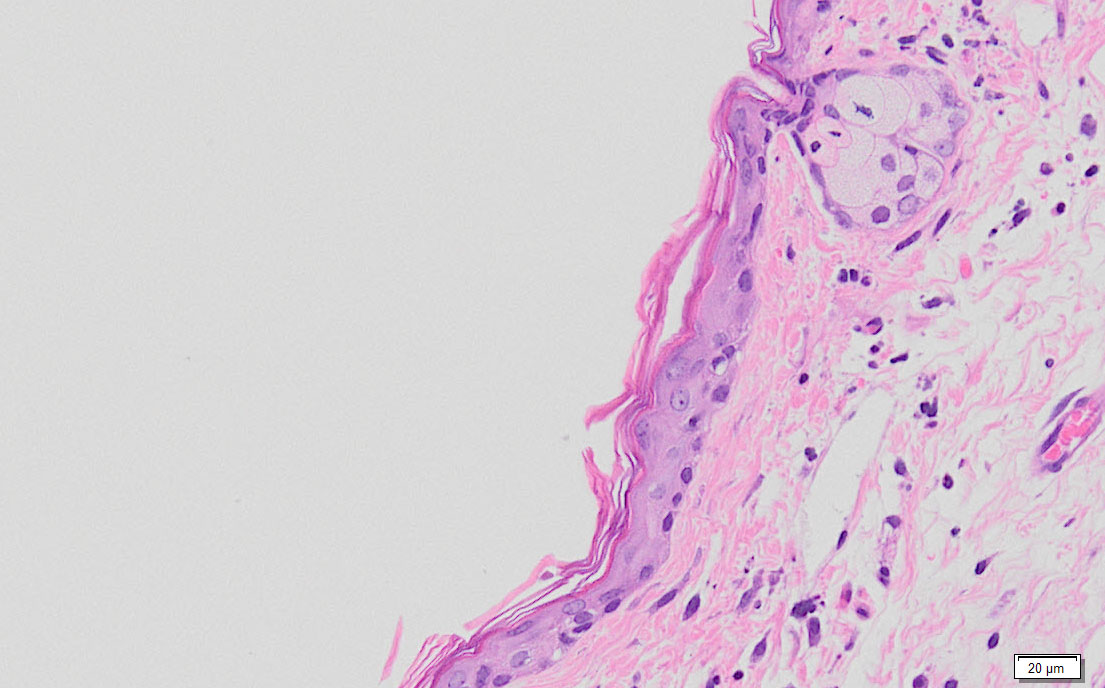

Supplement: Figure S1 [file peerj-04-1890-s003.zip › Raw data for Fig 3 A/0.16mg SM-3.jpg]

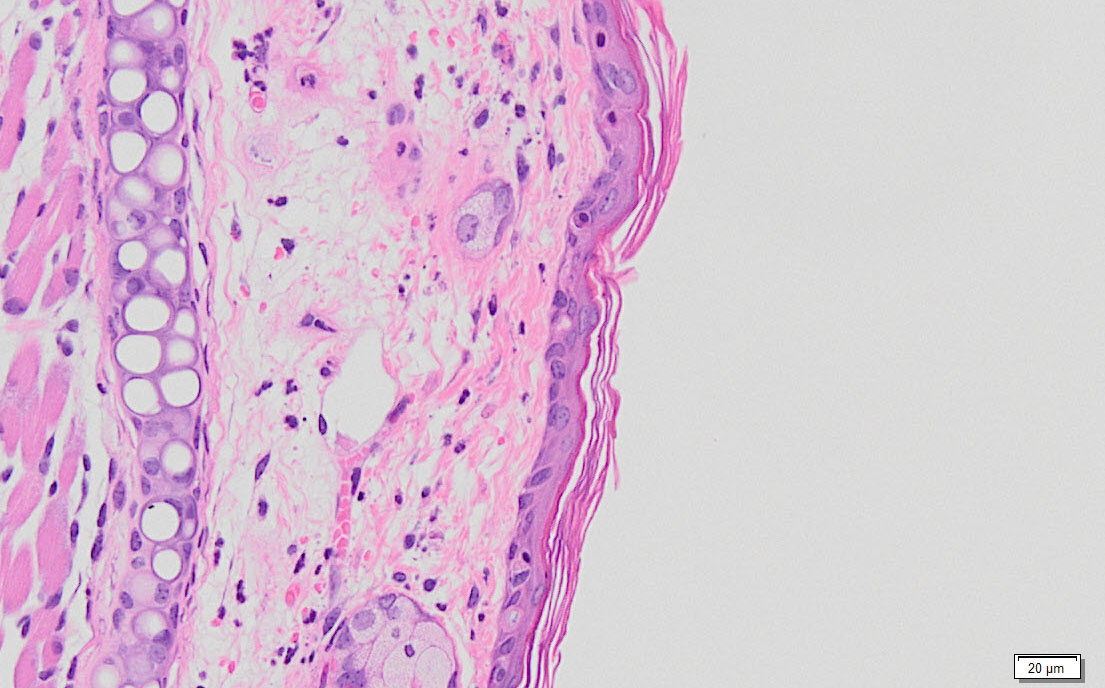

Supplement: Figure S1 [file peerj-04-1890-s003.zip › Raw data for Fig 3 A/0.16mg SM-4.jpg]

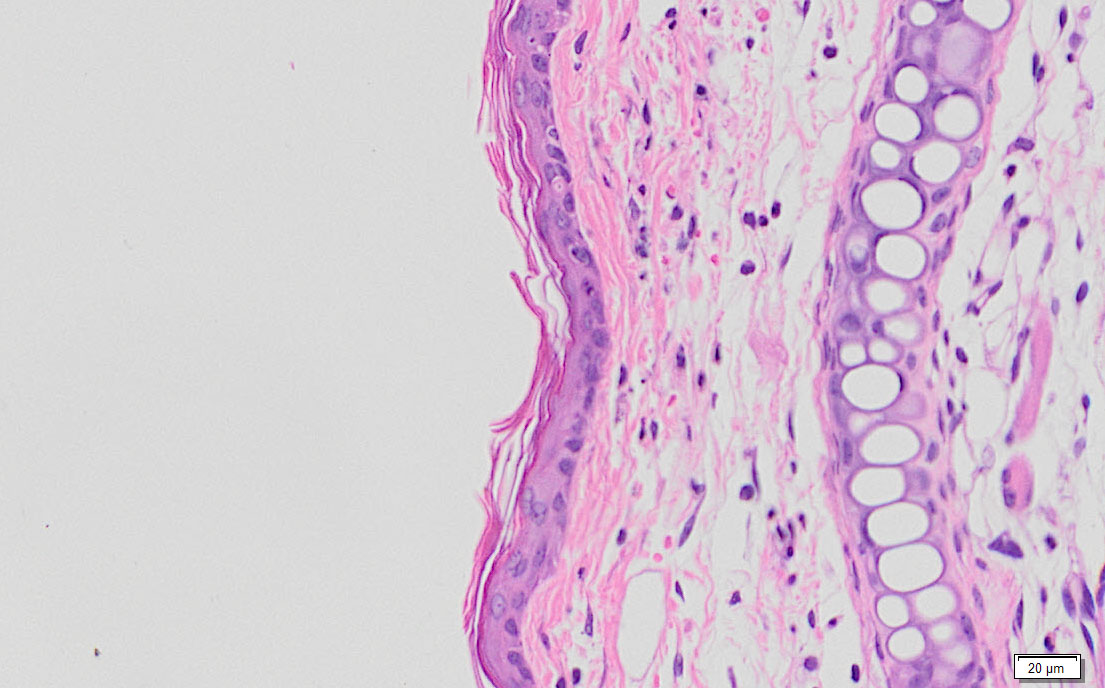

Supplement: Figure S1 [file peerj-04-1890-s003.zip › Raw data for Fig 3 A/0.16mg SM-5.jpg]

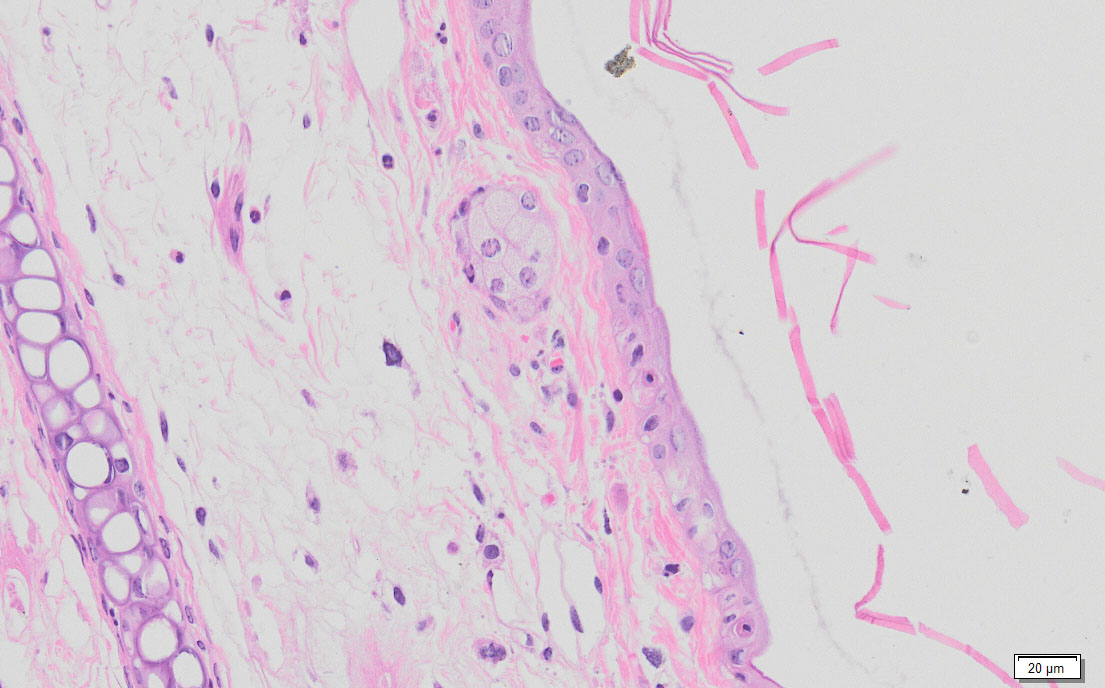

Supplement: Figure S1 [file peerj-04-1890-s003.zip › Raw data for Fig 3 A/0.64mg SM+ABT888-1.jpg]

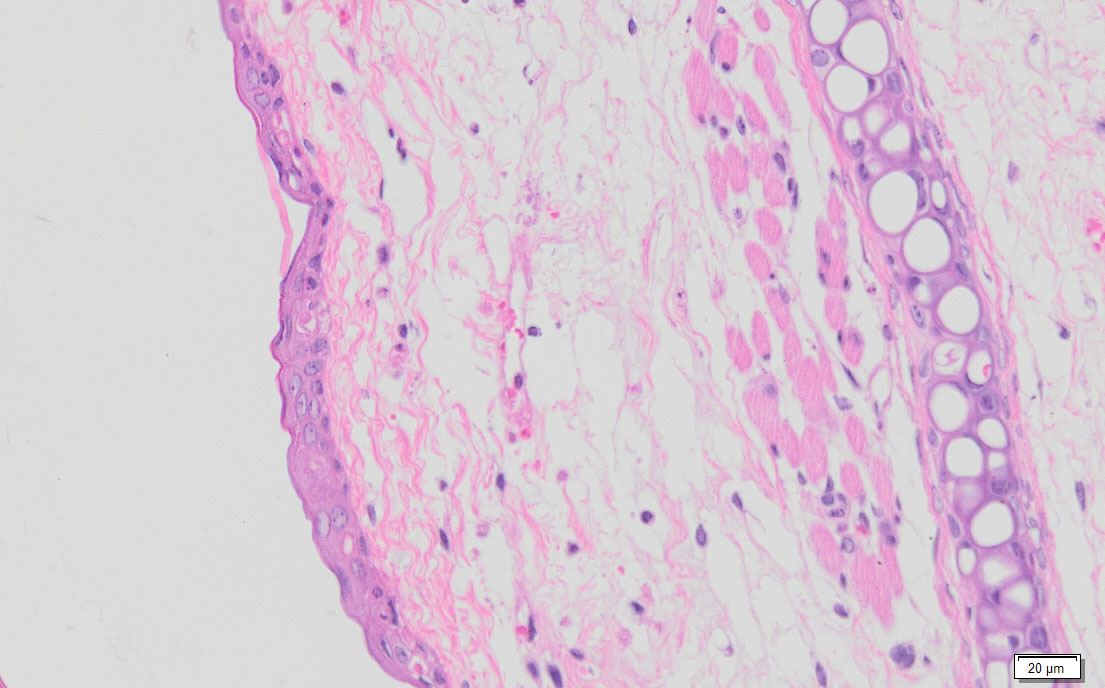

Supplement: Figure S1 [file peerj-04-1890-s003.zip › Raw data for Fig 3 A/0.64mg SM+ABT888-2.jpg]

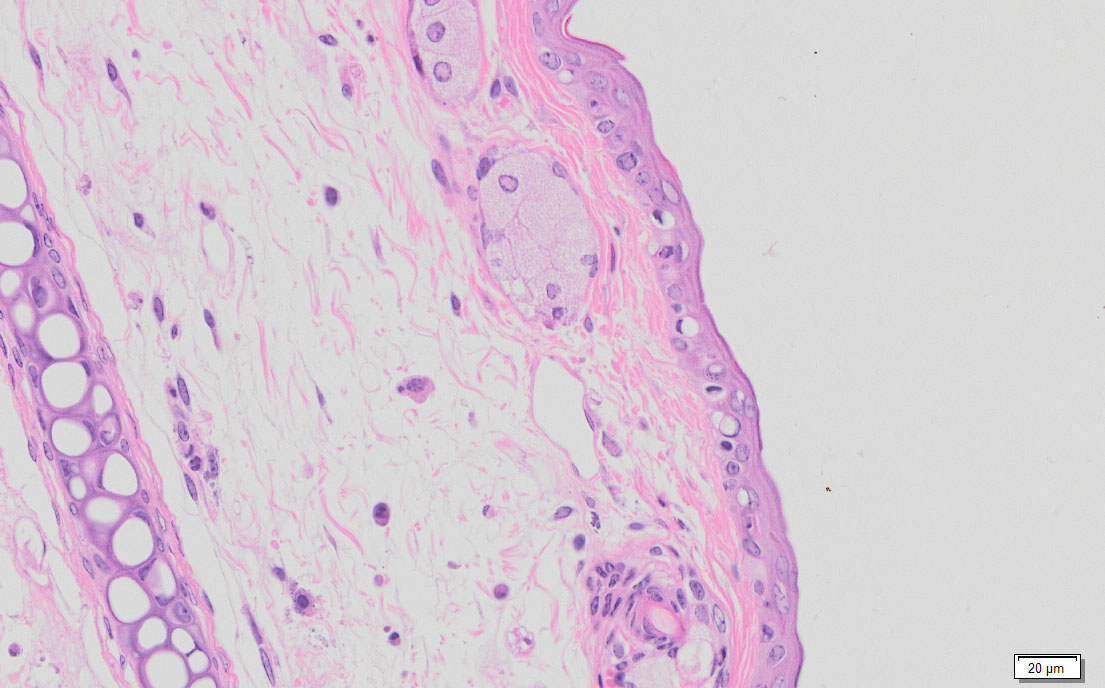

Supplement: Figure S1 [file peerj-04-1890-s003.zip › Raw data for Fig 3 A/0.64mg SM+ABT888-3.jpg]

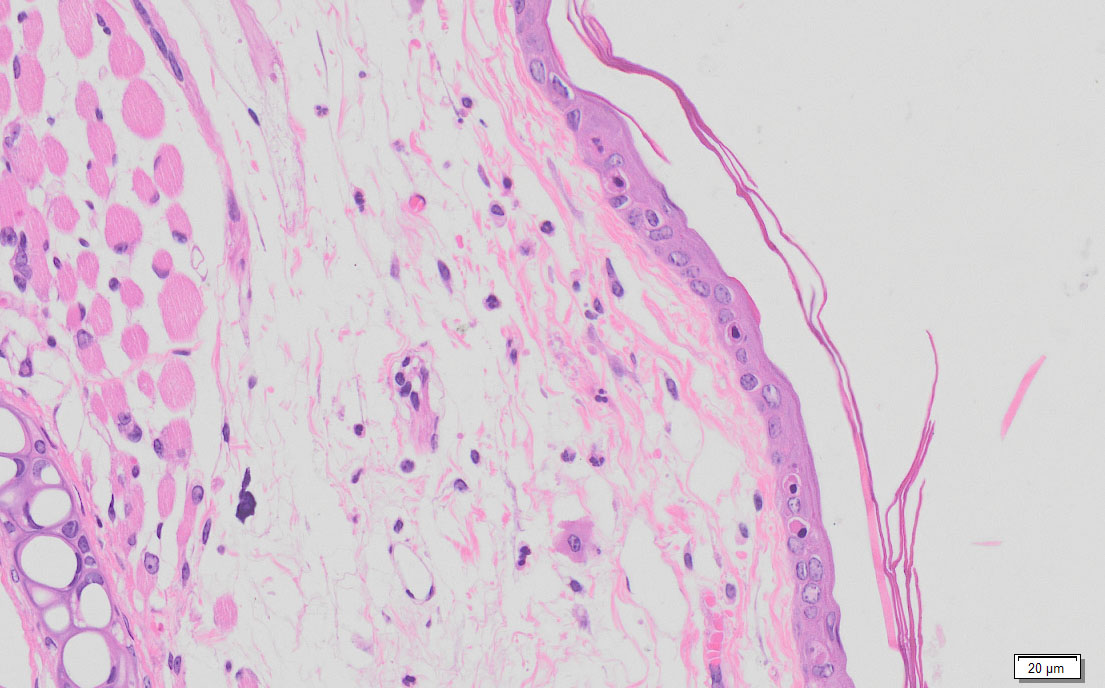

Supplement: Figure S1 [file peerj-04-1890-s003.zip › Raw data for Fig 3 A/0.64mg SM+ABT888-4.jpg]

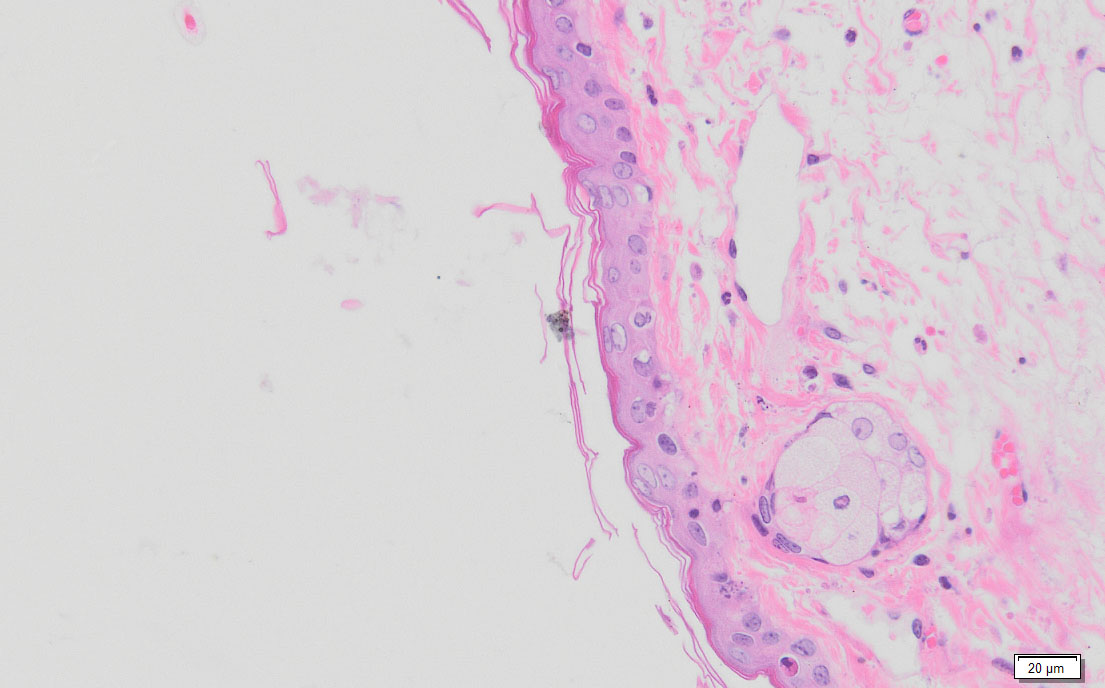

Supplement: Figure S1 [file peerj-04-1890-s003.zip › Raw data for Fig 3 A/0.64mg SM+ABT888-5.jpg]

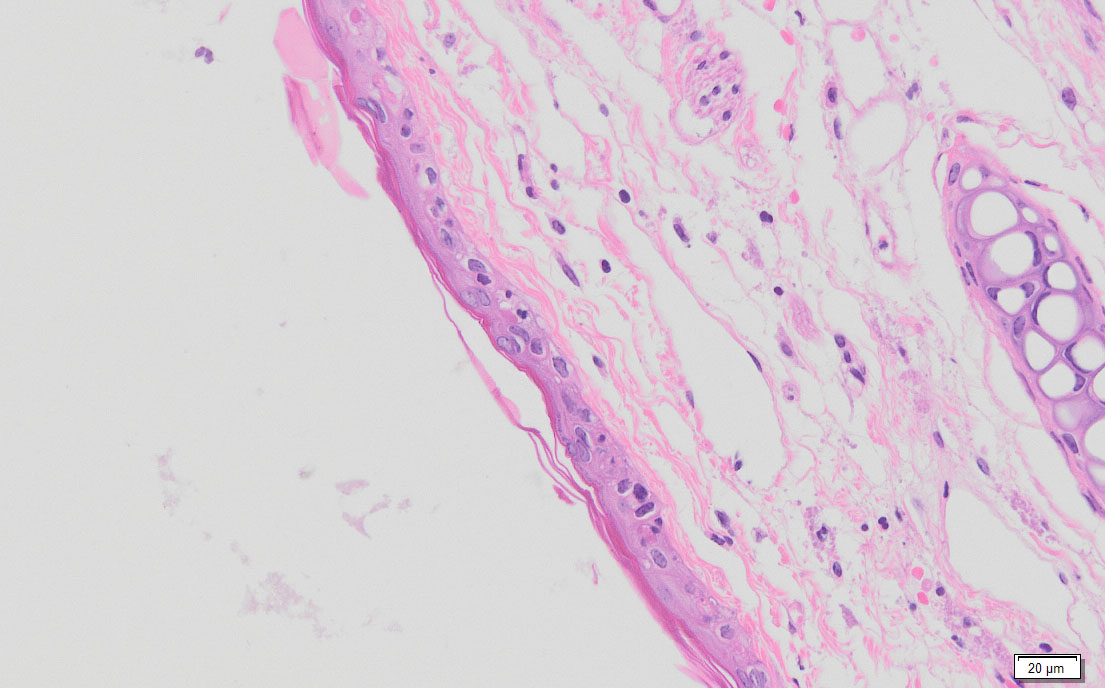

Supplement: Figure S1 [file peerj-04-1890-s003.zip › Raw data for Fig 3 A/0.64mg SM+ABT888-6.jpg]

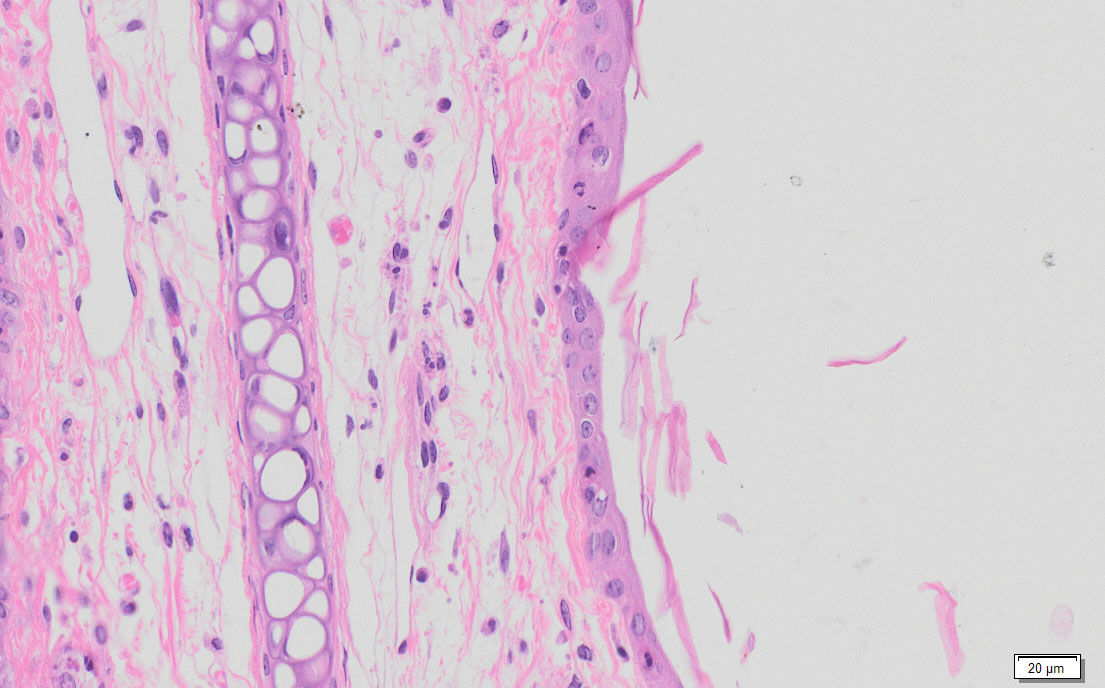

Supplement: Figure S1 [file peerj-04-1890-s003.zip › Raw data for Fig 3 A/0.64mg SM+ABT888-7.jpg]

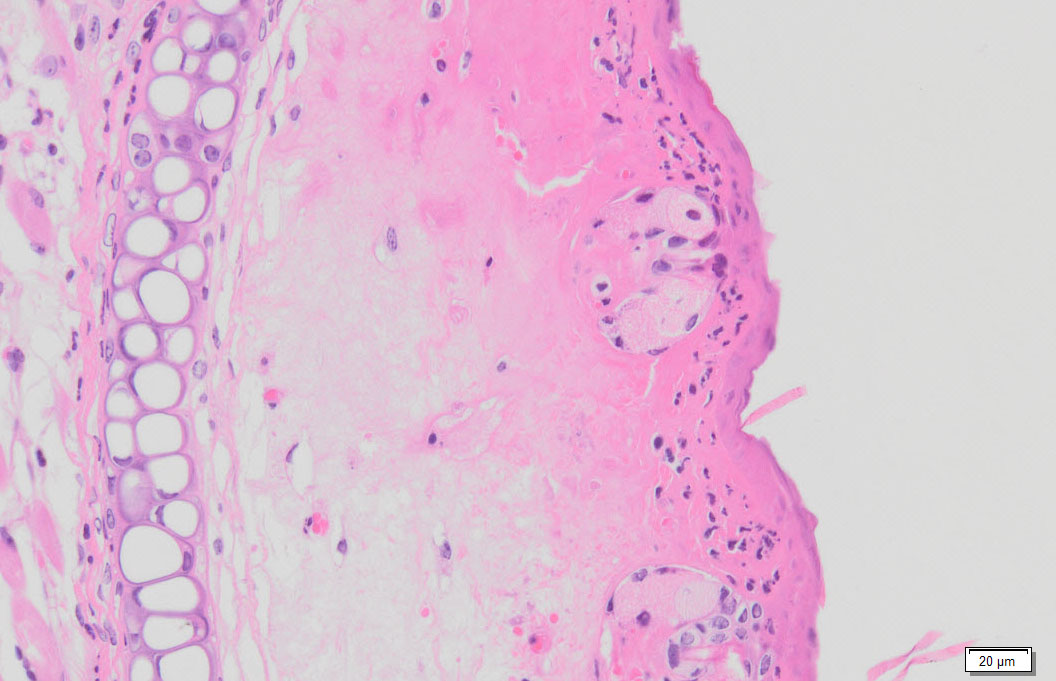

Supplement: Figure S1 [file peerj-04-1890-s003.zip › Raw data for Fig 3 A/0.64mg SM-1.jpg]

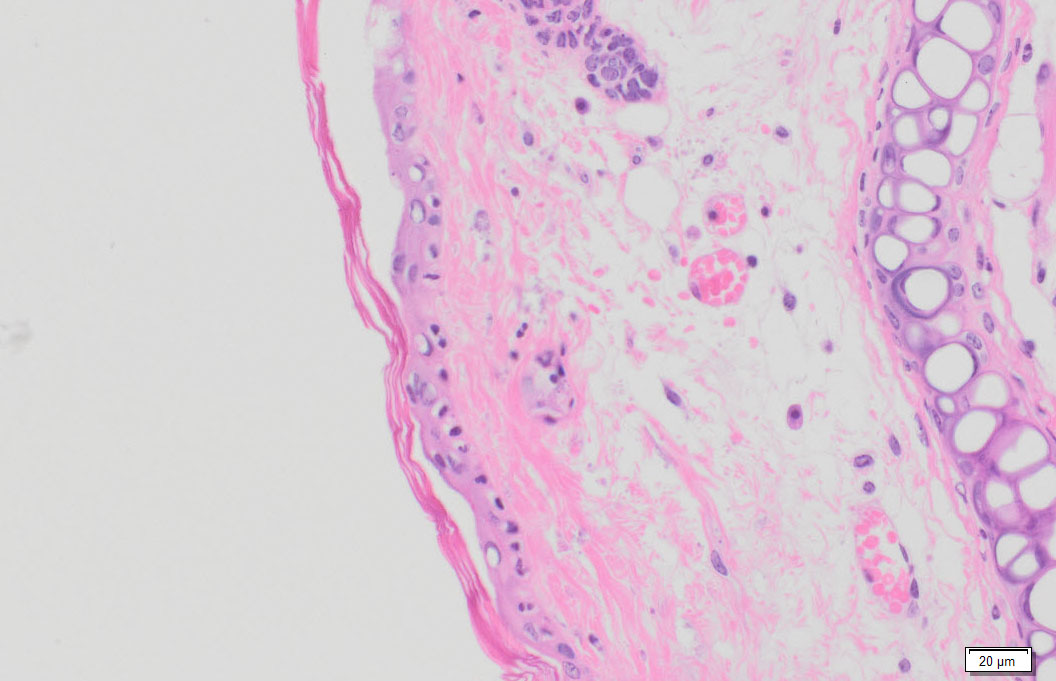

Supplement: Figure S1 [file peerj-04-1890-s003.zip › Raw data for Fig 3 A/0.64mg SM-2.jpg]

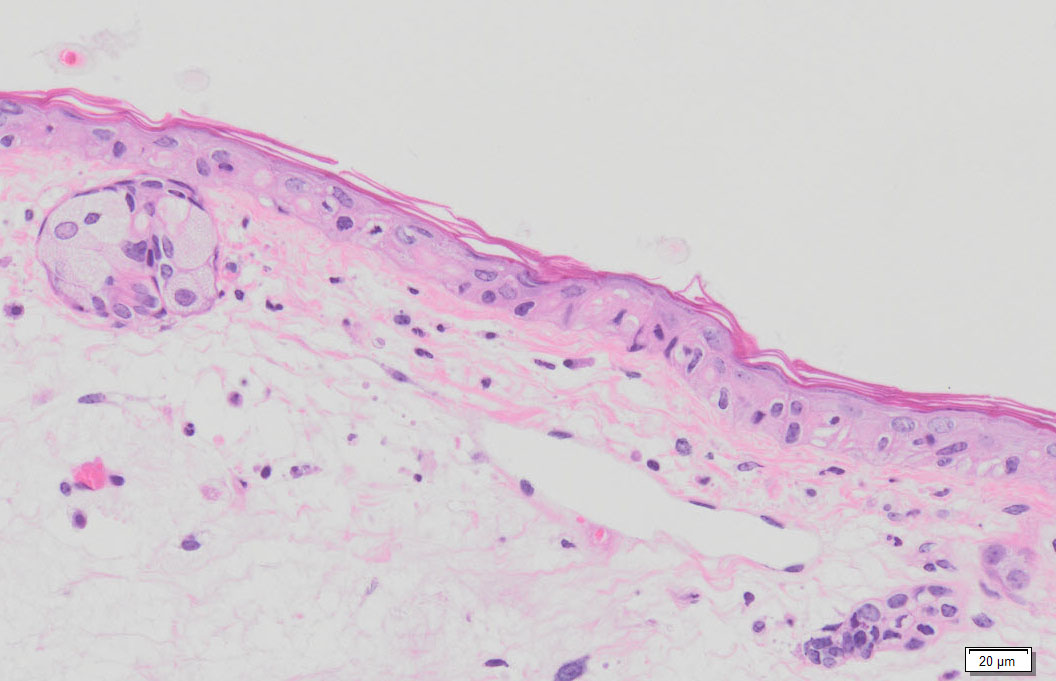

Supplement: Figure S1 [file peerj-04-1890-s003.zip › Raw data for Fig 3 A/0.64mg SM-3.jpg]

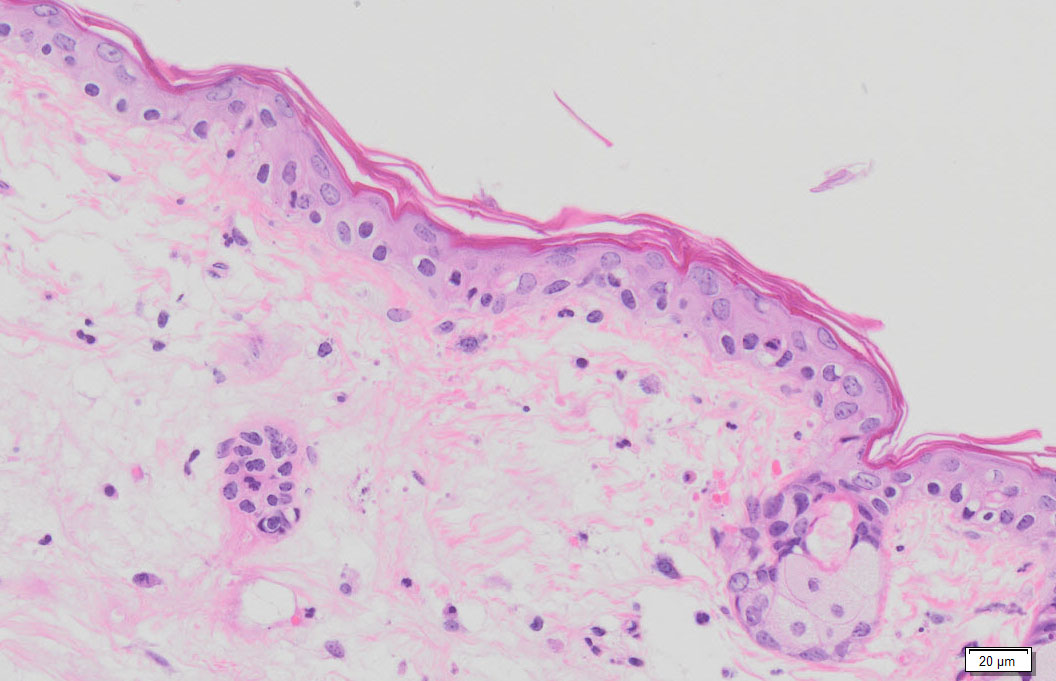

Supplement: Figure S1 [file peerj-04-1890-s003.zip › Raw data for Fig 3 A/0.64mg SM-4.jpg]

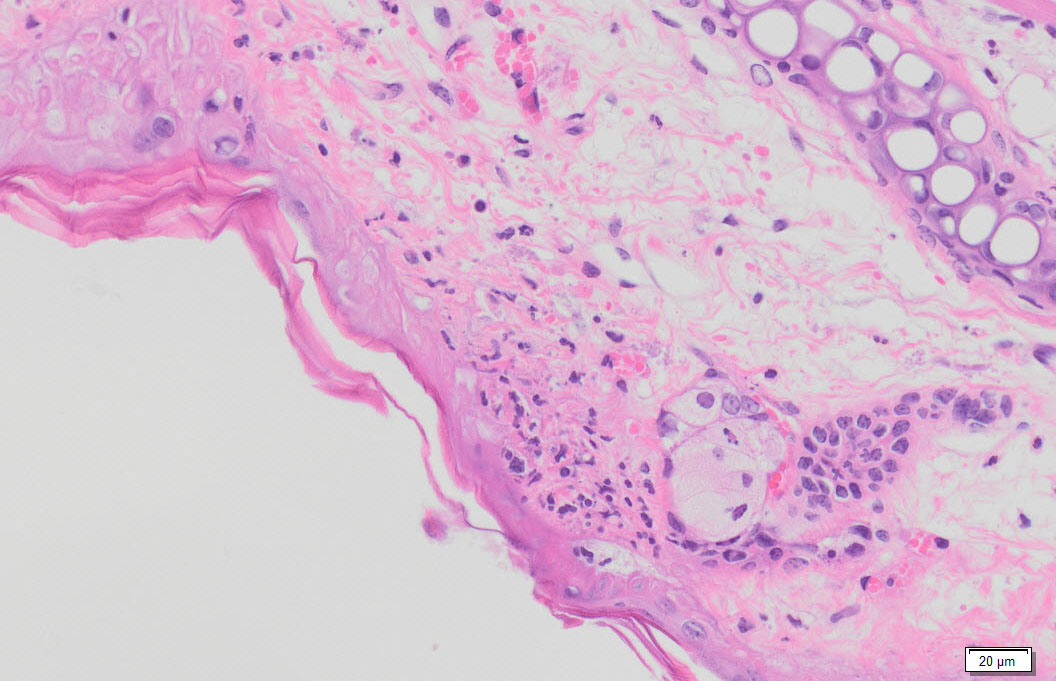

Supplement: Figure S1 [file peerj-04-1890-s003.zip › Raw data for Fig 3 A/0.64mg SM-5.jpg]

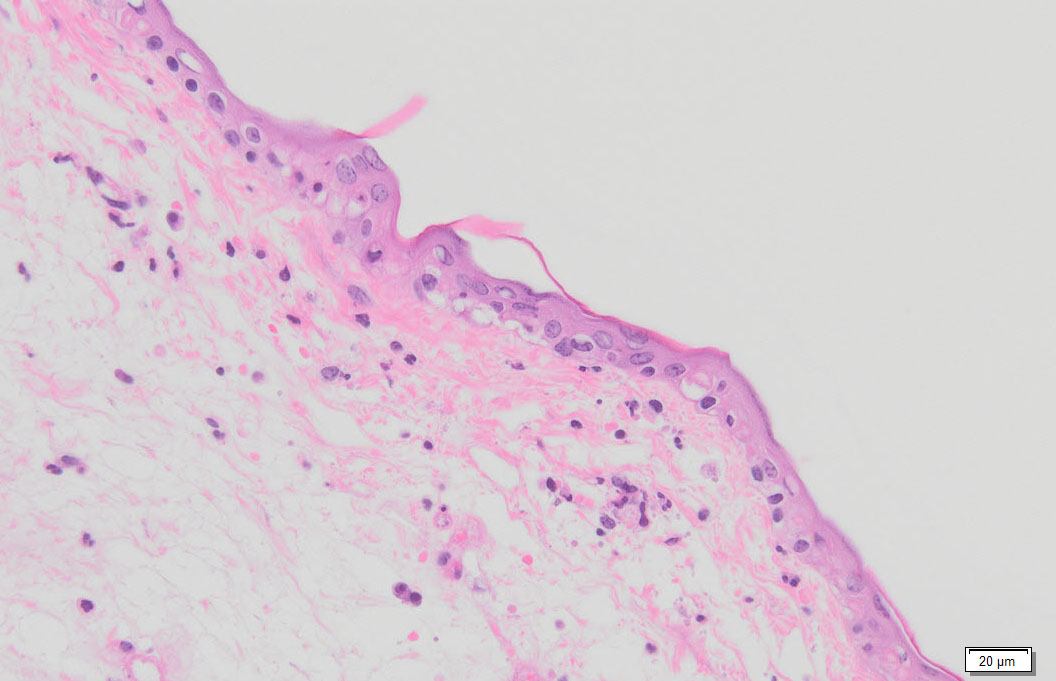

Supplement: Figure S1 [file peerj-04-1890-s003.zip › Raw data for Fig 3 A/0.64mg SM-6.jpg]

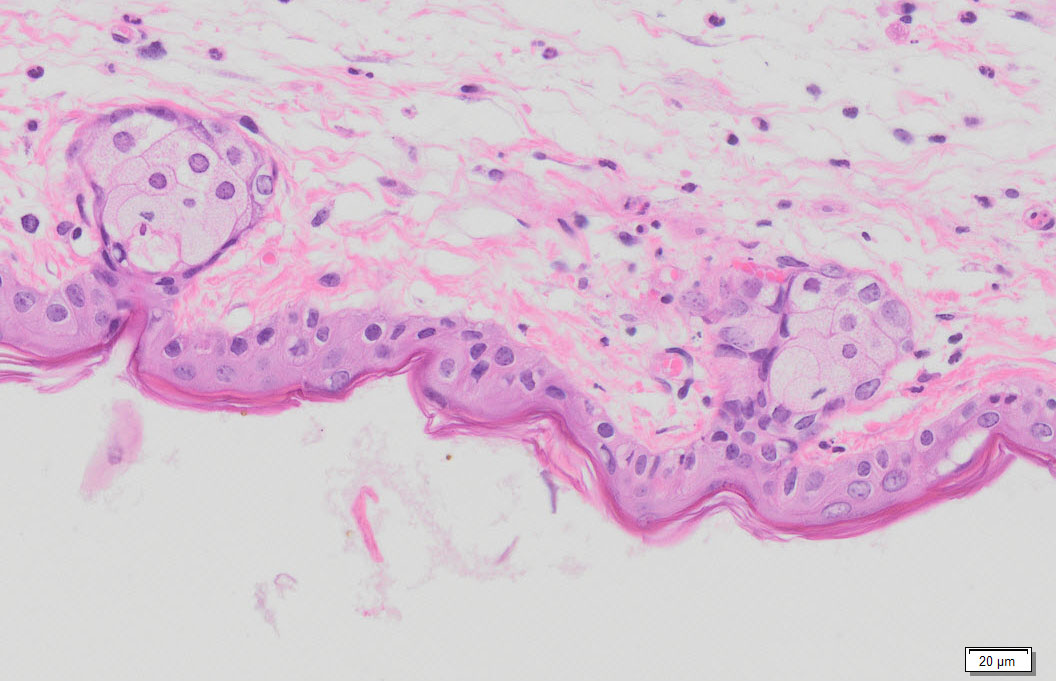

Supplement: Figure S1 [file peerj-04-1890-s003.zip › Raw data for Fig 3 A/0.64mg SM-7.jpg]

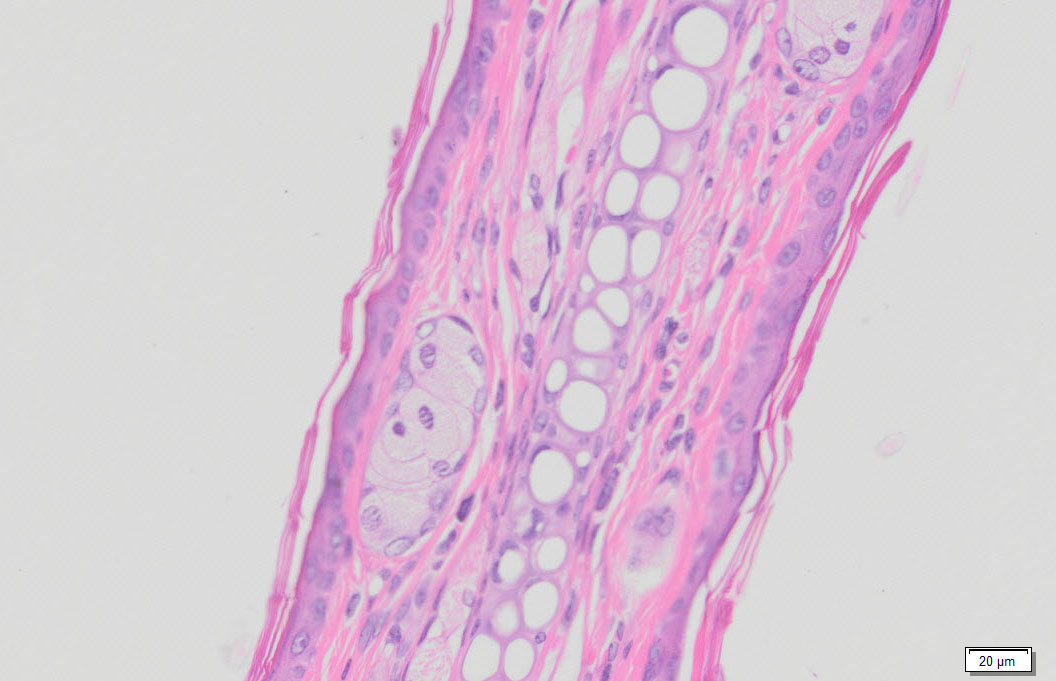

Supplement: Figure S1 [file peerj-04-1890-s003.zip › Raw data for Fig 3 A/con-1.jpg]

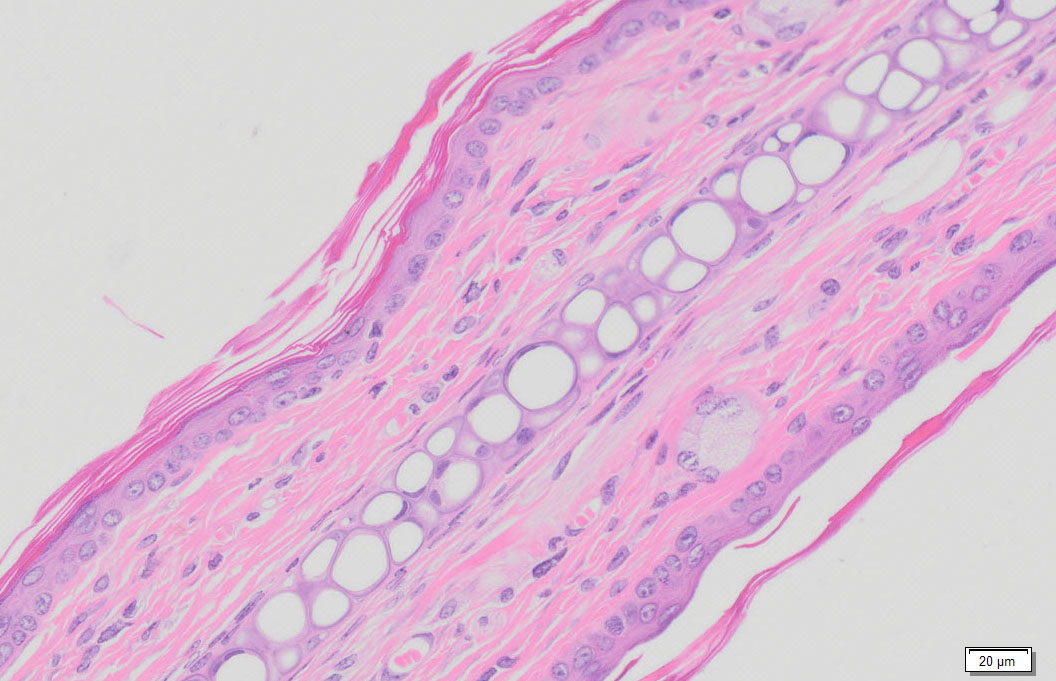

Supplement: Figure S1 [file peerj-04-1890-s003.zip › Raw data for Fig 3 A/con-2.jpg]

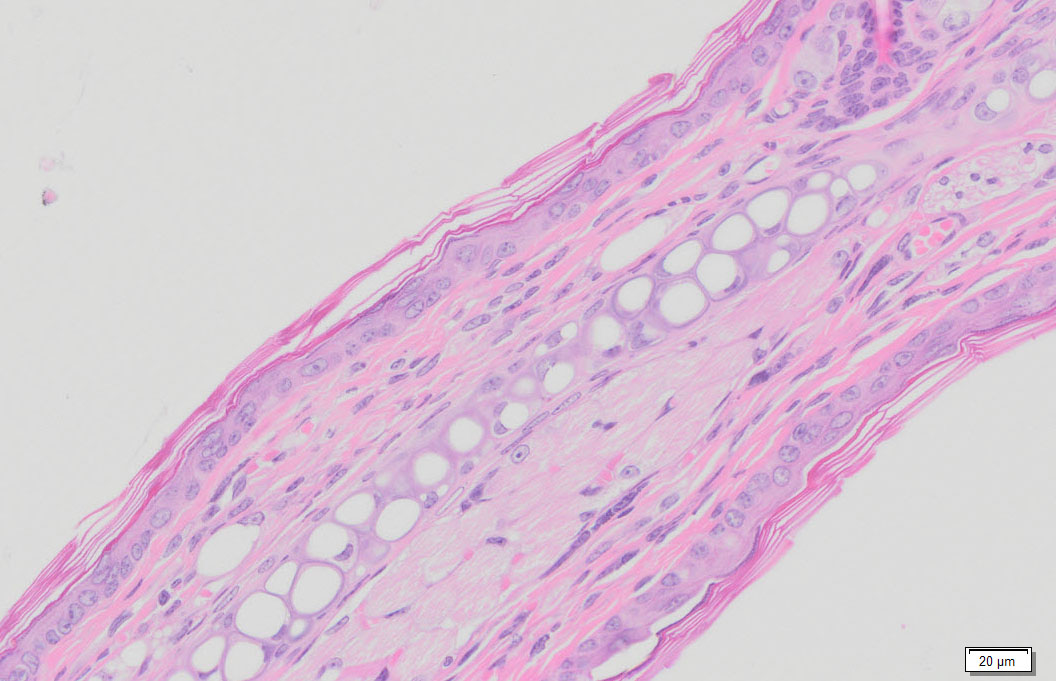

Supplement: Figure S1 [file peerj-04-1890-s003.zip › Raw data for Fig 3 A/con-3.jpg]

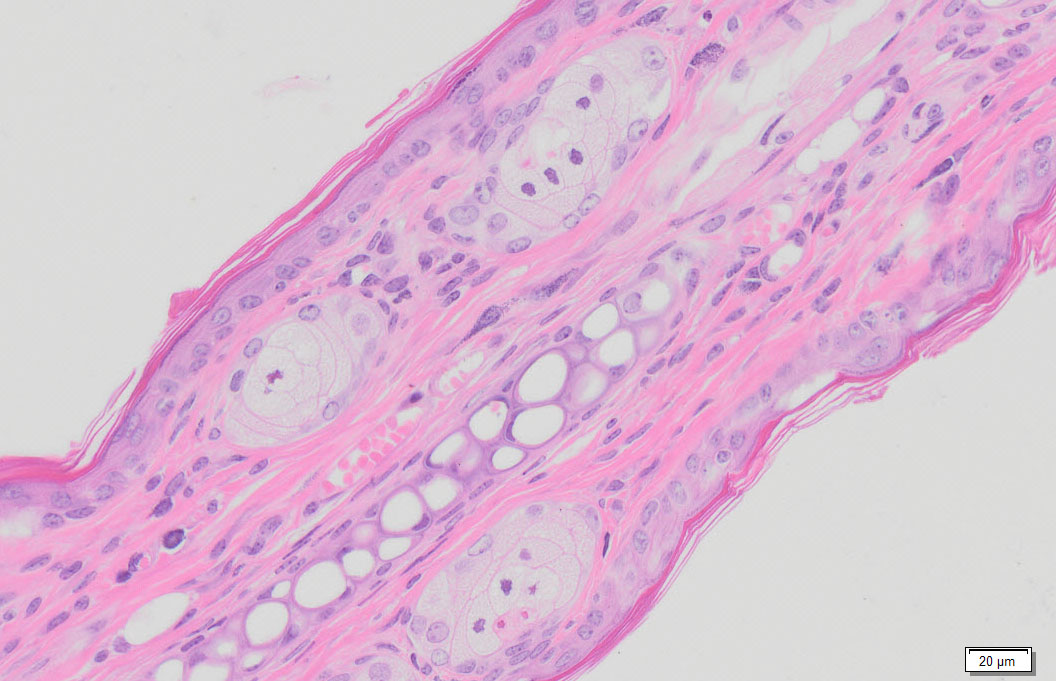

Supplement: Figure S1 [file peerj-04-1890-s003.zip › Raw data for Fig 3 A/con-4.jpg]

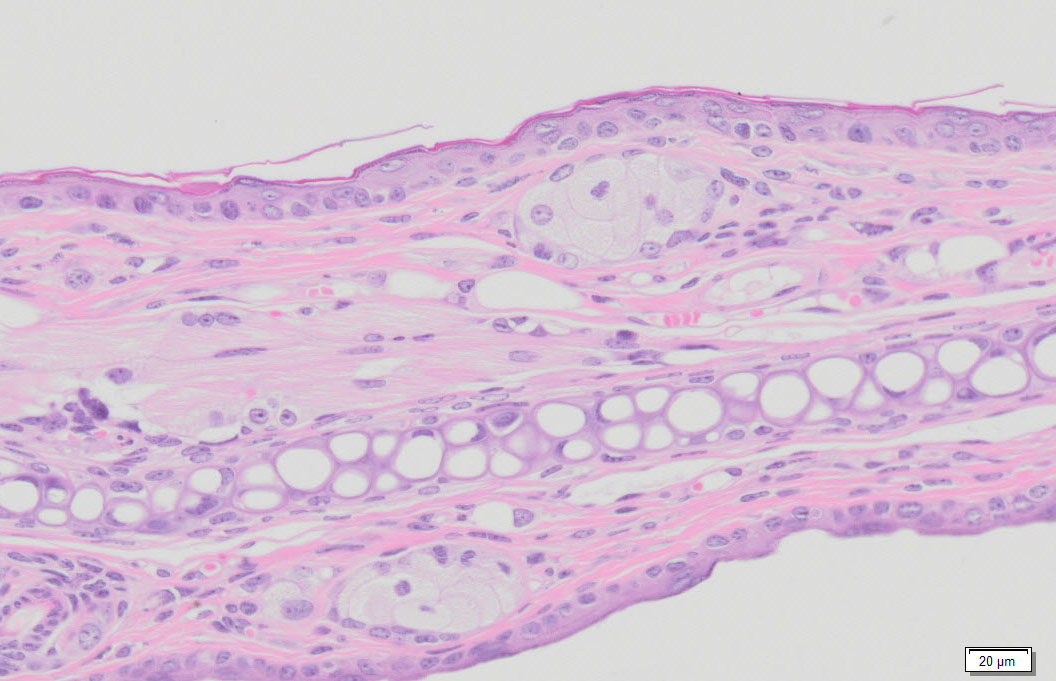

Supplement: Figure S1 [file peerj-04-1890-s003.zip › Raw data for Fig 3 A/con-5.jpg]
